# Surrogate endpoints in global health research: still searching for killer apps and silver bullets?

**DOI:** 10.1136/bmjgh-2018-000755

**Published:** 2018-03-08

**Authors:** Madhukar Pai, Samuel G Schumacher, Seye Abimbola

**Affiliations:** 1 McGill Global Health Programs and McGill International TB Centre, McGill University, Montreal, Quebec, Canada; 2 Foundation for Innovative New Diagnostics, Geneva, Switzerland; 3 The George Institute for Global Health, University of New South Wales, Sydney, New South Wales, Australia; 4 School of Public Health, University of Sydney, Sydney, New South Wales, Australia

**Keywords:** health systems, public health

In clinical research, there is widespread acceptance that surrogate endpoints may not translate to long-term benefits.^1–3^ Clinical epidemiologists highlight the hazards of surrogate measures (eg, biomarkers, laboratory test results and short-term improvements in health) that substitute for outcomes which are important for patients (eg, avoiding premature death or severe disability). For example, in cardiovascular research, improvements in parameters such as blood pressure or cholesterol may not improve outcomes such as deaths. Improvements in surrogate endpoints may not correlate with real outcomes of interest (and may even increase the risk of death, in some cases). And there are many examples and case studies in the literature that illustrate the hazards of using surrogates in clinical epidemiology.^1–3^

In comparison, in global health, we are often stunned when interventions that showed improvements in surrogate endpoints do not lead to lives being saved. Take, for example, the new tuberculosis (TB) detection technology, Xpert MTB/RIF(R) (Cepheid Inc, Sunnyvale, California, USA), an automated, molecular test for TB and drug resistance. Xpert MTB/RIF was first endorsed by WHO in 2010^4^ and has since been rolled out in many countries with over 23 million tests conducted in the past 6 years.^5^ While the test is rapid, accurate and much superior to tests that have been in use for decades,^6^ some pragmatic randomised controlled trials (RCTs) did not show improvements in long-term outcomes such as reduction in mortality.^7 8^ These results have prompted media headlines such as ‘improved diagnostics fail to halt the rise of tuberculosis.”^9^

The recent RCT in India of the WHO Safe Childbirth Checklist presents another example. The WHO Safe Childbirth Checklist is a quality-improvement tool to promote systematic adherence to practices that have been associated with improved childbirth outcomes.^10^ In a large-scale study in 24 districts in India, adherence of birth attendants to essential birth practices was higher in facilities that participated in the coaching-based WHO Safe Childbirth Checklist programme than in those that did not. But maternal and perinatal mortality and maternal morbidity did not differ significantly between the two groups.^10^ Again, this prompted media headlines such as ‘a birth checklist fails to reduce deaths in rural India’^11^ and ‘a lifesaving childbirth tool was successfully introduced in India—but saved no lives’.^12^

There are many more such examples in global health, from complex water and sanitation interventions, to TB vaccine trials, where surrogate endpoints do not align well with long-term outcomes.^13 14^ But given the weak health systems in many low-income and middle-income countries, it is surprising that global health researchers and journalists have great expectations that new tools, widgets, drones and checklists will save lives and are then stunned and disappointed when they do not. These ‘technological’ innovations often improve surrogate endpoints but may fail to meaningfully improve clinical outcomes in part because such outcomes improve only when a series of causal events are improved or completed. Often, the entire cascade of events in healthcare needs to improve; merely improving one or two steps (eg, diagnosis or process of care) may not lead to improvements in overall outcomes or result in sustained benefit.

In addition, there are innovations for which the expectation of improved health outcomes may not be necessary; especially innovations that aim to facilitate the patient–provider interface through improved coordination and integration of care (eg, using text message reminders, video consultations, remote monitoring and medication adherence technologies).^15–17^ For example, while a patient’s health may not improve simply because they are able to consult their general practitioner via Skype, such innovations may make the process and experience of care more convenient, save the costs of travel and forfeited work and reduce care-seeking delays. But again, important as they are, these benefits are only points in the causal cascade that link innovations to improved health outcomes, and indicators of these benefits (rather than health outcomes) may be sufficient to determine whether an innovation is effective.

**Figure 1:**
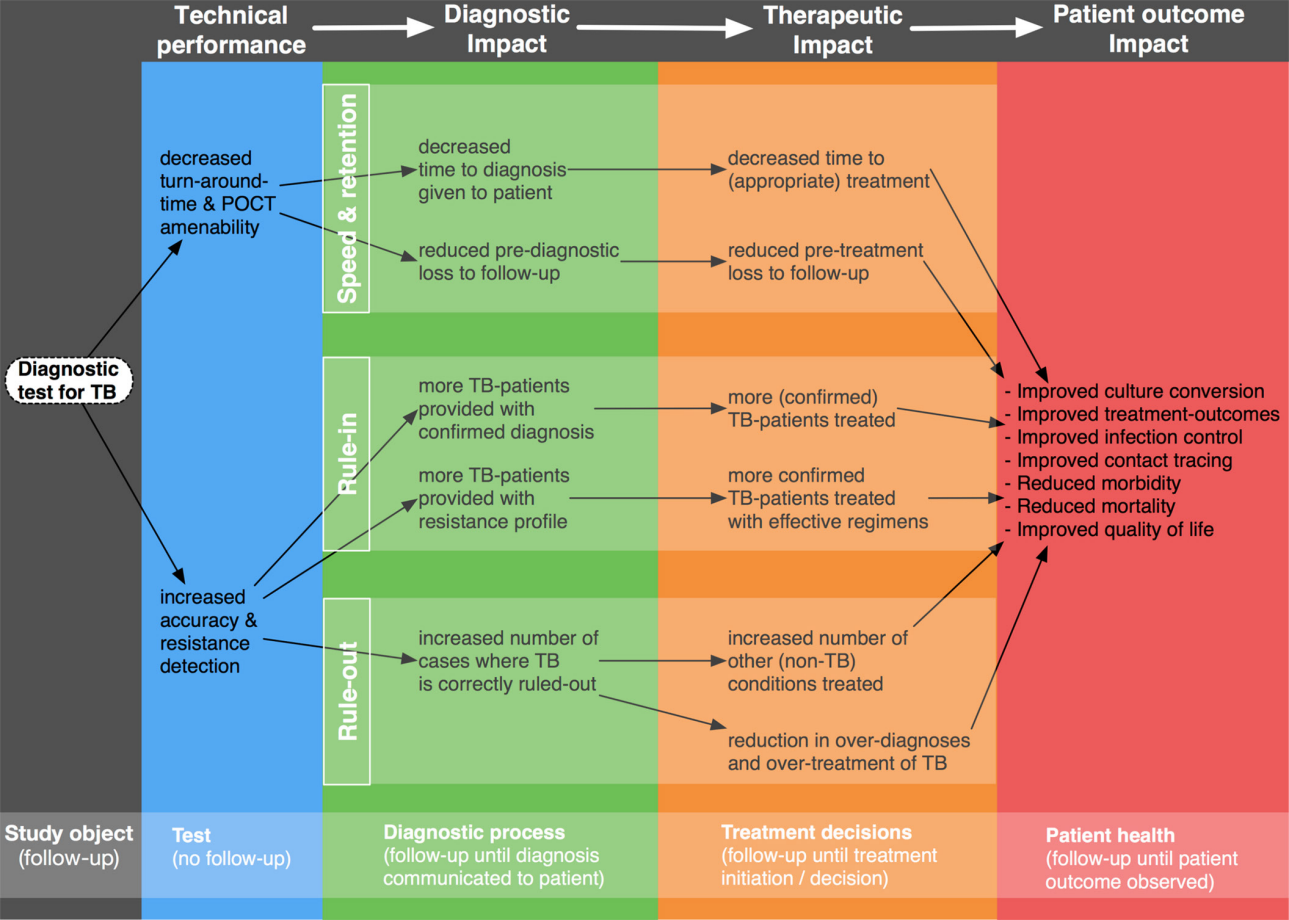
A framework for outlining the pathways through which new tuberculosis (TB) tests can result in improved patient outcomes. Source: Schumacher *et al*^18^ *PLoS ONE* 2016 (open access under Creative Commons license).

It is important that global health researchers are realistic when choosing indicators of effectiveness—an innovation designed to reduce costs or improve convenience should be evaluated primarily based on those indicators. For example, the purpose of a TB diagnostic test is to rapidly and accurately identify patients with TB. Once this is done, other factors become more prominent (see figure 1),^18^ for example, what treatment is initiated and why (empirical vs test and treat), how quickly, treatment completion rates and treatment of comorbidities. These steps in the care cascade are often weak in many settings.^7 19–21^ In that case, is it fair to expect a TB test to save lives? Likewise, it is not fair to expect that adherence to a childbirth checklist would save lives. The purpose of a checklist is to ensure that essential tasks are done during childbirth. But what if pregnant women do not come to health facilities on time, or when referred for urgent hospital care they are unable to reach hospitals, which may even lack facilities for Caesarean section or blood transfusion?^12^

We need to be more strategic about using surrogate endpoints in global health. First, because some innovations are developed essentially to influence such surrogate endpoints; second, because health system factors may predictably intervene in the care cascade and third because waiting for long-term outcomes could delay the introduction of useful innovations. On the other hand, we must not use surrogate endpoints naively, given the dangers inherent in such endpoints. We must learn from clinical epidemiologists who argue that, ‘researchers should avoid surrogate endpoints unless they have been validated’^2^ and caution us that ‘the use of surrogate outcomes should be limited to situations where a surrogate has demonstrated robust ability to predict meaningful benefits’.^3^

Global health researchers should design innovative studies to show if and how surrogate endpoints alter subsequent causal events or influence patient outcomes. If we care about reducing mortality after use of a TB test or a childbirth checklist, then we should also ensure that health systems are able to deliver subsequent life-saving activities in the care cascade; a shift from a fixation on tools to patient-centred solutions; from trials of standalone innovations to evaluations of complex, multisectoral health interventions. Such studies do not have to be large RCTs with mortality as the main outcome, given the methodological challenges of conducting RCTs, when (unlike for drug or vaccines) the effectiveness of the innovation being trialled (eg, a diagnostic, checklist, or text message reminder) depends on events further downstream in the care cascade, which in turn depend on health system context.^22–24^

RCTs may have little value in evaluating innovations such as the WHO Childbirth Checklist for which there is already strong and widely accepted evidence for the effectiveness of each of their component interventions.^10^ Even if an RCT were to show improved maternal and neonatal outcomes in one setting, it is unclear that the intervention would have had a similar effect elsewhere, given that implementation and health system context vary significantly.^25^ Indeed, causal pathways in public health interventions are often long and complex, and RCT results are subject to effect modification.^26^ Unfortunately, when such positive effects are found in RCTs, the result is often promoted as though the findings of the study would be applicable everywhere. And despite the limitations of RCTs or outcomes used in RCTs in global health, donors and guideline development groups (eg, Grading of Recommendations, Assessment, Development and Evaluations^27^ often prioritise evidence from RCTs, even when RCTs may not be necessary or appropriate for the innovation being considered for policy.

**Figure 2:**
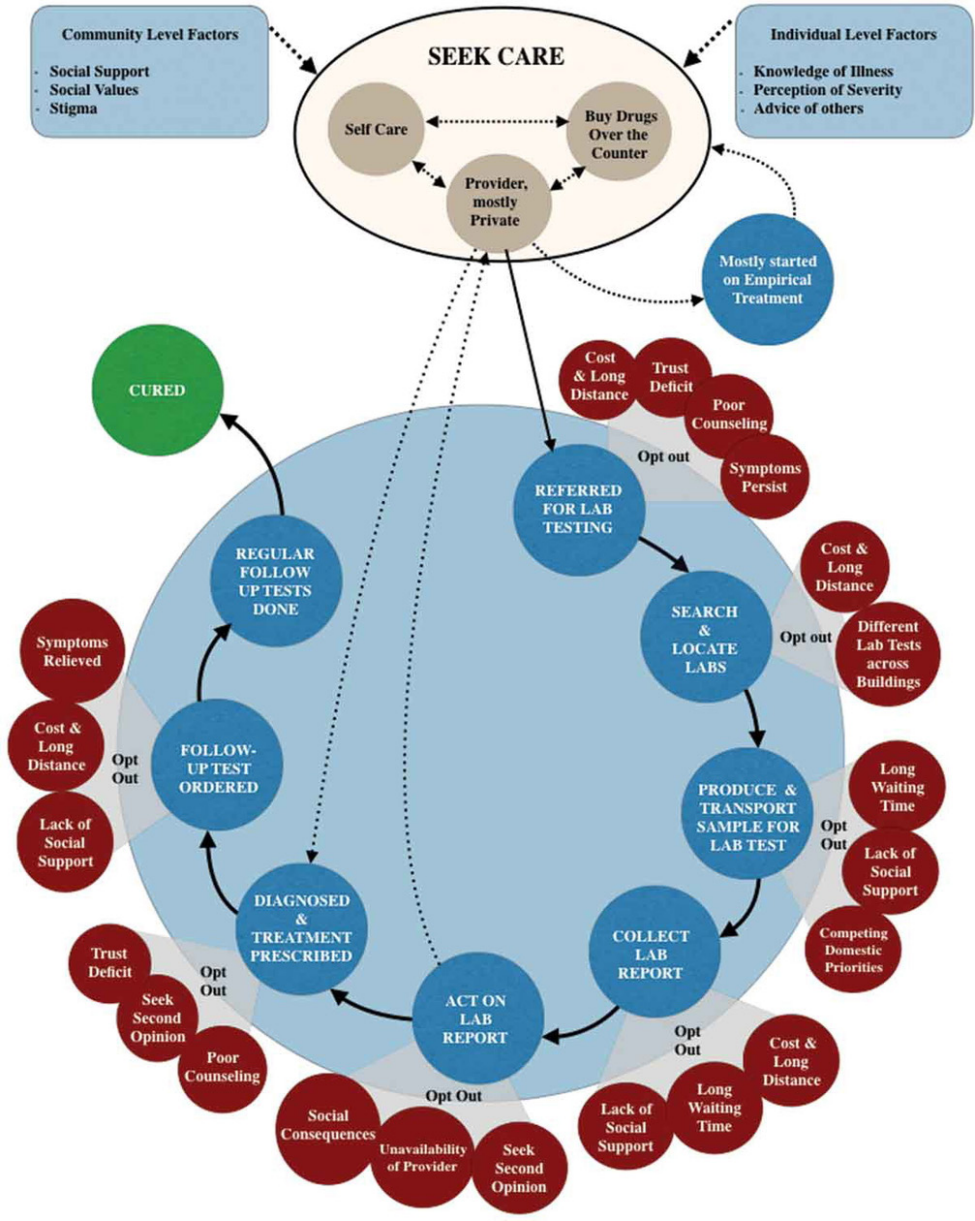
How patients navigate the diagnostic ecosystem in a fragmented health system in India. Source: Yellapa *et al*^28^ Global Health Action 2017 (open access under Creative Commons license).

We therefore propose two ways forward. First, map out the exact point in the cascade of care pathway in which an innovation is inserted and theorise how it may make a difference and what barriers may impede its effects on health outcomes. Using the TB example, while figure 1 shows a conceptual causal pathway through which a diagnostic can have an impact,^18^ figure 2 shows an actual, messy pathway that patients navigate within a real world, fragmented health system,^28^ thus identifying assumptions that must hold, and barriers that must be overcome, for a diagnostic test to fulfil its potential. Second, use theory-driven heath systems and implementation research^29 30^ on the adoption of innovations to confirm or refute assumptions of how an innovation might work along the mapped-out care pathway and examine the impact of innovations on the surrogate endpoints along the care cascade. Such implementation research can provide rich insights into how we can optimise the impact and transferability of innovations, depending on context.^31–33^

We need to explicitly lower unreasonable expectations of the impact of innovations, when surrogate endpoints are used, and when findings (including of RCTs) may not be transferable beyond specific and similar context. We need to explain the difference between surrogate endpoints and patient outcomes to policymakers and also to journalists to make sure their reporting is factual and honest. Neither the Xpert MTB/RIF test nor the WHO Safe Childbirth Checklist should be given up just because results of RCTs on their effect on mortality are not favourable. New tools have their place and are urgently needed in global health. Searching for silver bullets and killer apps are worthwhile endeavours, but we must not expect them to be ‘silver’ or ‘killer’ when introduced into systems that are suboptimal. If we care about making a real difference in global health, we also need to work on strengthening health systems to ensure holistic, effective and long-lasting solutions for patients and communities.
